# Glucose Metabolism and Tumor Microenvironment: Mechanistic Insights and Therapeutic Implications

**DOI:** 10.3390/ijms26051879

**Published:** 2025-02-22

**Authors:** Wiktoria Andryszkiewicz, Julia Gąsiorowska, Maja Kübler, Karolina Kublińska, Agata Pałkiewicz, Adam Wiatkowski, Urszula Szwedowicz, Anna Choromańska

**Affiliations:** 1 Faculty of Medicine, Wroclaw Medical University, Pasteura 1, 50-367 Wroclaw, Poland; wiktoria.andryszkiewicz@student.umw.edu.pl (W.A.); julia.gasiorowska@student.umw.edu.pl (J.G.); maja.kubler@student.umw.edu.pl (M.K.); karolina.kublinska@student.umw.edu.pl (K.K.); agata.palkiewicz@student.umw.edu.pl (A.P.); adam.wiatkowski@student.umw.edu.pl (A.W.); 2 Department of Molecular and Cellular Biology, Faculty of Pharmacy, Wroclaw Medical University, Borowska 211A, 50-556 Wroclaw, Poland; urszula.szwedowicz@umw.edu.pl

**Keywords:** glycolysis, the Warburg effect, phosphofructokinase, lactate dehydrogenase A, hexokinase, pyruvate kinase, tumor microenvironment, cancer stem cells, tumor-associated macrophages

## Abstract

Metabolic reprogramming in cancer cells involves changes in glucose metabolism, glutamine utilization, and lipid production, as well as promoting increased cell proliferation, survival, and immune resistance by altering the tumor microenvironment. Our study analyzes metabolic reprogramming in neoplastically transformed cells, focusing on changes in glucose metabolism, glutaminolysis, and lipid synthesis. Moreover, we discuss the therapeutic potential of targeting cancer metabolism, focusing on key enzymes involved in glycolysis, the pentose phosphate pathway, and amino acid metabolism, including lactate dehydrogenase A, hexokinase, phosphofructokinase and others. The review also highlights challenges such as metabolic heterogeneity, adaptability, and the need for personalized therapies to overcome resistance and minimize adverse effects in cancer treatment. This review underscores the significance of comprehending metabolic reprogramming in cancer cells to engineer targeted therapies, personalize treatment methodologies, and surmount challenges, including metabolic plasticity and therapeutic resistance.

## 1. Introduction

In neoplastically transformed cells, genetic mutations occur, resulting in metabolic reprogramming. It enables continued proliferation, growth, and differentiation, thereby increasing the cell’s chances of survival and further dissemination. Moreover, the cancer cell’s metabolism has a significant impact on the regulation of the antitumor immune response [1,2]. Among the primary processes undergoing reprogramming are glucose metabolism, fatty acid metabolism, glutamine metabolism, as well as pathways that directly modulate the tumor microenvironment [3]. The alteration of glucose metabolism provides a competitive advantage to cancer cells, both over surrounding stromal cells and immune cells [1,2]. Increased glucose uptake in cancer cells allows them to outcompete other cells for limited metabolic resources [2,3]. Furthermore, the intensified process of glycolysis leads to lactate overproduction, which modifies the tumor microenvironment and subsequently impairs immune surveillance [3]. Another metabolic process that undergoes modification in cancer cells is glutaminolysis, which occurs at significantly higher rates. Glutamine becomes a key nitrogen and carbon source for anabolic processes, supplying the necessary precursors for cellular growth and proliferation. Additionally, glutamine contributes to energy production in catabolic pathways and is involved in cellular signaling. Deregulation of lipid metabolism is one of the most critical alterations in cancer cells, primarily characterized by enhanced lipid synthesis. Modifying the Krebs cycle provides substrates for synthesizing fatty acids and cholesterol, which are essential for forming cellular membranes, producing energy, and synthesizing bioactive signaling molecules. This metabolic reprogramming enables cancer cells to generate and store energy and support their regeneration and continued proliferation. Understanding the metabolism of cancer cells and its impact on their functionality is crucial for cancer diagnosis and developing more effective therapeutic strategies [3,4]. Moreover, it provides a critical foundation for the development and application of novel, more targeted therapies.

## 2. Key Mechanisms of Cancer Cell Metabolism

### 2.1. The Warburg Effect

The origins of the Warburg effect sub-study can be traced back to the early 20th century, when German biochemist Otto Warburg demonstrated that cancer cells prefer anaerobic respiration over aerobic respiration, compared to normal cells. During lactate fermentation, even in the presence of oxygen, cancer cells convert large amounts of glucose to lactate and reduce oxidative phosphorylation [5,6,7]. This reaction provides cancer cells with high levels of lactate production, in exchange for faster though less efficient ATP production. The effect thus involves a shift from oxidative phosphorylation to glycolysis or lactate fermentation [8,9]. The biochemical process was designated the Warburg effect or aerobic glycolysis by the researcher in question, who proposed that the underlying cause of this phenomenon is damage to mitochondria [9,10]. Nevertheless, this assertion was subsequently invalidated, with the proposal that aerobic glycolysis is more advantageous for the proliferation of these cells. This metabolic pathway also necessitates cellular adaptation, which can be achieved through the increased expression of several glycolytic enzymes, leading to augmented glycolysis: phosphofructokinase 1 (PFK1), phosphofructokinase 2 (PFK2), hexokinase (HK) and lactate dehydrogenase A (LDHA) [11]. This active metabolic process is caused by the reprogramming of proto-oncogenes, which results in the blocking of cell differentiation and the facilitation of oncogenesis, in addition to the alteration of signaling pathways. It has been demonstrated through scientific investigation that a single post-transcriptional alteration to the pyruvate kinase muscle isoform 1 (PKM1), which plays a pivotal role in glycolysis, results in the formation of the pyruvate kinase muscle isoform 2 (PKM2). The formation of this isoform is a prerequisite for the redirection of cellular metabolism towards the aerobic glycolysis pathway, which is a critical step in the process of tumor formation. PKM2 is regarded as a metabolic switch, exerting a pivotal influence on the Warburg effect. This protein plays a pivotal regulatory role, as evidenced by the fact that reducing its catalytic activity is linked to tumor progression and the development of the Warburg effect. A reduction in PKM2 activity results in its substrate phosphoenolpyruvate (PEP) accumulation, inhibiting the glycolytic enzyme triosephosphate isomerase (Tpi). This inhibition results in the activation of the pentose cycle, which constitutes a supplementary pathway to glycolysis and is responsible for the induction of the aforementioned effect and promotion of tumor growth [9].

Nevertheless, the Warburg effect does not provide a comprehensive explanation for the mechanisms underlying the excessive cell proliferation and growth of cancer cell masses due to aerobic glycolysis. This can be attributed to giving glucose alone, not guaranteeing the essential components required for cell growth, including sulfur, nitrogen, and phosphorus. Thus, the multicellular mass, i.e., the tumor, is not supplied with sufficient oxygen and nutrients, which results in cancer-inducing angiogenesis. This is an imperfect and inefficient solution; thus, the metabolic switch, or Warburg effect, is designed to meet the energy or construction needs of the rapidly proliferating tumor cells [12,13]. In addition to the previously mentioned glucose, glutamine is an important energy source and anabolic fuel that enables the delivery of tricarboxylic acid cycle (TCA cycle) intermediates to other biosynthetic pathways. Furthermore, it is essential to highlight that cancer cell growth depends on glutamine. Without this amino acid, cells undergo rapid death [13].

A hypothesis concerning tumor metabolism was put forth a few years ago. This hypothesis suggested that mitochondrial respiration occurs aerobically, thereby providing evidence that the effect described occurs mainly in stromal fibroblasts. In this theory, primarily fibroblasts, through their catabolic processes, provide cancer cells with high-energy compounds, including lactate, ketones, and glutamine, which serve as the biofuel for the fusion reaction. Providing substrates to cancer cells enables the production of substantial quantities of ATP through aerobic respiration. In this manner, their growth and development are enabled, which is tantamount to tumor progression. This hypothesis has been designated the reverse Warburg effect [9]. Nevertheless, contemporary research into cancer cell metabolism indicates that the Warburg effect is not merely a process of ATP production but a multifaceted phenomenon. Indeed, it has been demonstrated that 95% of the ATP generated is essential for maintaining vital cellular functions rather than contributing to proliferation [12].

### 2.2. Oncogenic Signals and Metabolic Reprogramming

**Figure 1:**
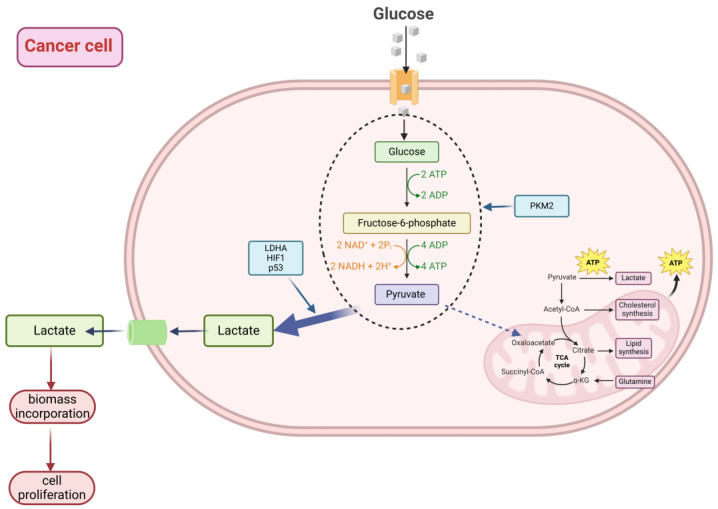
Diagram illustrating the Warburg effect. The Warburg effect, also known as aerobic glycolysis, involves the conversion of glucose into lactate while bypassing the mitochondria and avoiding oxidative phosphorylation. This process is regulated by factors such as pyruvate kinase M2 (PKM2), lactate dehydrogenase A (LDHA), hypoxia-inducible factor-1 (HIF-1), and the tumor suppressor protein p53. Created in BioRender. Szwedowicz, U. (2025) [8,9,10,11,12,13].

It has been demonstrated that mutations significantly influence the metabolic enzyme activity and aerobic glycolysis of tumors. These mutations may result in the induction of oncogenes or the inactivation of tumor suppressors, which can considerably impact cellular metabolism. Oncogenic mutations can influence cellular metabolism, including alterations in phosphatase and tensin homolog (PTEN), phosphatidylinositol 3-kinase (PI3K), and the oncogenic transcription factor Myc and p53. Modifications to the PI3K pathway frequently significantly influence tumor proliferation and survival. In addition to PI3K activation, the AKT pathway is activated, stabilizing hypoxia-inducible factor 1 (HIF-1). PI3K contributes to the loss of PTEN by antagonizing this tumor suppressor, leading to increased glycolysis by activating AKT and HIF-1 (Figure 1). AKT also plays a pivotal role in stimulating glycolysis by increasing its expression, phosphorylating glycolytic enzymes such as PFK and HK, and enhancing glucose translocation across the cell membrane. Additionally, it causes the activation of the mechanistic target of rapamycin (mTOR), which indirectly affects other metabolic pathways through the activation of HIF-1. The activation of mTOR is also affected by Ras mutation through the PI3K-Akt-mTOR signaling pathway. HIF-1, conversely, is implicated in modulating several factors, including c-Met, erythropoietin, platelet-derived growth factor-b, glucose transporter 1 (GLUT1), and transforming growth factor-a. This influences cellular activities, including angiogenesis, glycolysis, and cell survival. Furthermore, pyruvate dehydrogenase kinase is activated by HIF-1, which in turn inactivates pyruvate dehydrogenase and obstructs the flow of pyruvate into the aforementioned TCA cycle [8]. A reduction in pyruvate transport into the mitochondria leads to a decline in the rate of oxidative phosphorylation and a corresponding decrease in oxygen consumption [8,11].

Conversely, Myc is implicated in the direct transcriptional activation of glycolytic enzymes, which impacts cell metabolism. Additionally, it cooperates with HIF-1 in the activation of certain glucose transporters, LDHA and PDK1, as well as glycolytic enzymes [14]. In contrast to the roles of AKT and HIF-1, Myc may also be involved in the regulation of genes related to glutamine metabolism [8].

## 3. Impact of the Tumor Microenvironment

The tumor cannot survive without its microenvironment. In addition to cancer cells, there are many other non-tumorigenic cells that form tumor microenvironments (TME). TME plays a vital role in tumor progression as it promotes the development of progenitor cells, tumor-initiating cells (TICs), and cancer stem cells (CSCs) also called tumor-initiating cells [15]. CSCs can be characterized as malignant, self-renewing, and pluripotent cells, which can unrestrictedly increase their number. The level of proliferation is low due to their low mitotic activity. Still, it decreases the tumor sensitivity to chemotherapy and radiation as these treatment methods target more actively proliferating cells [16]. What is more, CSCs play a crucial role in cancer cell plasticity and neoplastic formation as they allow cancer cells to avoid apoptosis, immune surveillance, and limitation of tumor growth [17]. CSCs were found in many solid tumors such as colorectal cancer, breast cancer and prostate cancer [18]. It is worth adding that the other components of TME, such as carcinoma-associated fibroblasts (CAFs) and tumor-infiltrating mesenchymal stem cells (MSCs) interact with CSCs and increase their population by promoting the conversion of cancer cells to malignant CSCs [19].

CAFs play a significant role in tumorigenesis. The study proved that CAFs promote tumor growth and vascularization. The researchers injected both the HPV16-derived skin carcinoma cell line and PDSC5 cells into mice with controlled fibroblasts and CAFs from HPV dysplastic skin. The results show that the tumor in mice injected with CAFs was more vascularized and grew faster than in mice injected with PDSC5 cells alone. Moreover, one reason for increased vascularization is the proangiogenic macrophages that CAFs recruited. It is worth noting that CAFs contribute to the recruitment of inflammatory cells, which is essential as the inflammation supports tumorigenesis and metastasis [20,21].

On the other hand, MSCs can interact with cancer cells and their environment and differentiate into CAFs [22]. MSCs are responsible for the recruitment of tumor-associated macrophages (TAMs) [23]. Macrophages play a significant role in tumor progression, as they can have pro- or anti-tumor roles. They can switch their phenotype under different conditions and become M1 or M2 macrophages. The M1 macrophages are protective against tumor growth compared to the M2 macrophages that support tumor progression. TAMs are described as M2 or M2-like phenotypes [24,25]. MSCs recruit TAMs via chemokines such as CCR2 and CCL2 [25,26]. Moreover, MSCs are responsible for the production of chemokines and cytokines. Under the influence of interleukin-1α (IL-1α) and interleukin-1β (IL-1β), MSCs produce prostaglandin E2 (PGE2), resulting in increased cyclooxygenase-2 (COX-2) expression resulting in cancer progression and its drug resistance [27,28]. Moreover, IL-6 produced by MSCs increases CSCs expressing CD133 in colorectal cancer cells leading to tumor progression [15]. It is also worth noting that MSCs’ and CSCs’ populations participate in tumor neovascularization. The secretion of proangiogenic factors such as IL-6 angiopoietin-1 and endothelin-1 (ET-1) support vessel formation [23].

Another vital cell surrounding the cancer is tumor-associated fibroblasts (TAF). TAF is essential in tumor progression and metastasis and differs from other fibroblasts by their matrix synthesizing capability as they are able to produce more factors that support tumor growth and angiogenesis including vascular endothelial growth factor (VEGF), alpha-smooth muscle actin (α-SMA), growth factor-beta (TGF-β) and basic fibroblast growth factor (bFGF) [29]. What is more, TAF blocks the immune responses by inhibiting the penetration of T lymphocytes [30]. It is worth mentioning that various nutrients can definitely alter cancer metabolism, one of which is oxygen. Low doses of oxygen (hypoxia) are one of the TME stresses that tumor cells must overcome to grow and expand. The hypoxia-inducible factors (HIFs), genetic depletion of acetyl-CoA carboxylase (ACACA or ACC1) or ATP citrate lyase (ACLY) offer protection from apoptosis caused by hypoxia. Firstly, HIFs promote glycolysis by targeting glycolytic genes under hypoxia and leading to their expression and therefore promote the nonoxidative forms of ATP production. They also enhance glucose catabolism by promoting the expression of glucose transporters [31]. Secondly, ACC1 is responsible for conversion of acetyl-CoA to malonyl-CoA in lipogenesis. Researchers silenced ACLY and observed increased levels of ACC1 leading to inhibition of lipogenesis as the production of acetyl-CoA and oxaloacetate from citrate was blocked. It leads to the conclusion that the inhibition of lipogenesis offers protection from apoptosis under hypoxia as the lipogenesis requires a great amount of oxygen and energy leading to glucose deprivation and matrix detachment. What is more, the depletion of ACLY or ACC1 reduces levels of oncogenic transcription factor ETV4. The decreased levels of ETV4, ACC1 or ACLY activates a so-called “anti-apoptotic expression program” promoting the activation of negative regulators of apoptosis as well as the suppression of genes that promote apoptosis [32,33].

Glucose uptake is also significant as it limits cancer growth and expansion. It is worth adding that competition between cancer cells and leukocytes may lead to decreased immune surveillance as they both need glucose to function properly. Due to tumors’ high metabolic rate, they overcompensate with T-cells, leading to poor cytokine production and lack of antigen recognition, allowing the tumor cells to proliferate [34]. Moreover, tumors adapt to hypoxia by enhancing glycolysis, increasing lactic acid production [35]. The study showed that higher serum levels of lactic acid correlate with decreased proliferation of human cytotoxic T lymphocytes (CTLs) and the suppression of cytokine production. The researchers stimulated CTLs with PMA/ionomycin and lactic acid, showing reduced production of IL-2 and IFN-γ. Decreased levels of intracellular perforin and granzyme-B were also observed, which proved even more that lactic acid suppresses cytotoxic immune activity [36].

## 4. Implications for Cancer Therapy

### 4.1. Therapeutic Targeting of Metabolism

Essential targets in therapeutic targeting of cancer metabolism are glycolytic enzymes, including LDHA, PKM2, HK2, ALDOA, G6PD, PPP cycle, glutamine serine metabolism and pyruvate oxidation (Table 1). LDHA plays a crucial role in cancer cell metabolism. LDHA catalyzes the conversion of pyruvate to lactate, the last step of glycolysis [37]. The elevated levels of this enzyme influence the development of many malignant characteristics in tumors with LDHA overexpression [38,39]. The hampering of LDHA has been observed to suppress cell proliferation, migration, tumor growth, and angiogenesis. Several studies have confirmed its anticancer efficacy concerning various cancers, including hepatocellular carcinoma (HCC), melanoma, osteosarcoma, non-small-cell lung cancer, and many others [38,40,41,42,43]. Furthermore, the inhibition of LDHA rarely shows toxicity to normal cells. The only adverse effects experienced by patients lacking LDHA are muscle rigidity and sudden myoglobinuria after tremendous physical activity. In conclusion, due to its high antitumor efficacy and lack of severe adverse effects, LDHA represents a promising potential antitumor target [38].

Pyruvate kinase (PK) is another defining enzyme in the glycolytic pathway. It catalyzes the final rate-limiting step of glycolysis, an irreversible transphosphorylation reaction, converting PEP and adenosine diphosphate (ADP) into pyruvate and ATP [44,45,46]. This review focuses on PKM2, predominantly expressed in cancer cells, among the four PK isoforms. What distinguishes PKM2 from other isoforms’ ability to switch between a highly active tetrameric form and a less active dimeric form that supports oncogenic processes by slowing down glycolysis to accumulate precursor biomolecules necessary for cancer biosynthesis pathways [44,45]. Because of its unique properties, PKM2 is a promising target for cancer therapy. Two main strategies were investigated: inhibition of its enzymatic activity or stabilization of the catalytically active tetrameric form [44,45,47]. Various small molecule inhibitors and activators have been developed to modulate PKM2 activity. For example, shikonin reduces glycolysis in cancer cells, but its use in the clinic is limited due to its toxicity and poor solubility. The efficacy of shikonin was evaluated in a study on esophageal cancer. In vitro, shikonin reduced the expression of PKM2, EGFR, PI3K, p-AKT, and HIF1α, which led to decreased viability of cancer cells, cell cycle arrest and apoptosis. In vivo, in mouse models with esophageal tumors, the use of shikonin resulted in a significant reduction in tumor mass [48]. Similarly, metformin inhibits PKM2 expression and increases the efficacy of chemotherapy. In a study conducted on MDAMB-231 breast cancer cells, metformin significantly reduced cell viability to 42.6% after 24 h. It also caused a 5.60-fold decrease in glucose levels and a 9.56-fold increase in reactive oxygen species (ROS), which contributed to apoptotic cell death. These effects were further enhanced by the application of electrical pulses [49]. Other compounds, such as proanthocyanidin B2, curcumin, and resveratrol, have demonstrated anticancer properties through PKM2 inhibition. Conversely, activators of PKM2 stabilize its tetrameric form, curtailing cancer cell metabolic flexibility and reducing tumor growth [44,47]. These dual approaches—using inhibitors and activators—underline PKM2′s versatility as a therapeutic target. However, inhibiting PKM2 does not always significantly suppress tumor growth, as cancer cells can activate alternative pathways to sustain biosynthesis. Interestingly, some studies propose that maintaining PKM2 in its active tetrameric state might block tumor biosynthesis by continuously driving glycolysis. Still, the long-term effects of sustained ATP production in cancer cells remain uncertain [47,50]. In conclusion, PKM2 plays a multifaceted role in cancer progression. Its distinctive characteristics provide an attractive target for both inhibitors and activators. However, challenges such as tumor adaptability highlight the necessity for further research to refine and optimize therapeutic strategies.

Hexokinase 2 (HK2) is overexpressed in most human cancers and, as a glycolytic enzyme, significantly impacts cancer metabolism [51,52,53]. Studies showed that inhibition of HK2 suppresses the proliferation of various cancers, for instance, pancreatic ductal adenocarcinoma (PDAC) and colorectal cancer (CRC) [43,54]. In CRC, HK2 inhibition diminishes the epithelial-to-mesenchymal transition (EMT), which plays a defining role in cancer metastasis. Consequently, targeting HK2 can be a promising method used in anticancer therapy. Additionally, its inhibition showed minimal adverse events in animal models, which suggests that this approach may also be safe for potential patients [53,54]. Inhibitors of HK2, such as lonidamine (LND), metformin, 2-deoxyglucose (2-DG), and 3-bromopyruvate (3-BrPA), are widely investigated. Many of these compounds are widely used in experimental studies. 2-DG, however, is associated with several adverse effects that unintentionally activate survival pathways in cancer cells, leading to its withdrawal from clinical use as an antitumor agent. Studies have shown that 2-DG disrupts the interaction between insulin-like growth factor 1 (IGF-1) and its binding protein IGFBP3, leading to the release of free IGF-1 and activation of the IGF1R receptor. As a result, signaling pathways such as PI3K/AKT and MEK-ERK are activated, which induce cell proliferation, apoptosis resistance, and cancer cell survival. Inhibition of IGF1R reduces these prosurvival effects, potentially increasing cancer cell sensitivity to 2-DG and enhancing its therapeutic potential [55]. Many HK2 inhibitors do not exhibit high antitumor efficacy as standalone agents. LND and metformin are promising chemosensitizers when combined with other chemotherapeutic drugs to achieve higher efficacy in cancer treatment. Other HK2 inhibitors, such as VDA-1102, also show promising results. VDA-1102, used as an ointment for the treatment of actinic keratosis (AK), a precursor to cutaneous squamous cell carcinoma (cSCC), has completed Phase II clinical trials, which assessed its efficacy and safety. [51,52]. Targeting HK2 exhibits high potential in cancer therapies; therefore, further investigation is required [56,57,58].

Aldolase A (ALDOA) is a glycolytic enzyme that produces glyceraldehyde-3-phosphate and dihydroxyacetone phosphate from fructose 1,6-bisphosphate. This is an essential step of glycolysis, which provides cells with energy [57,58]. It mainly concerns cancer cells, which rely on glycolysis to support their rapid growth and survival [59,60]. Studies show that ALDOA is overexpressed in several cancers, including HCC, prostate cancer (PCa), and lung adenocarcinoma. Moreover, its overexpression is consistent with increased tumor growth and metastasis, which leads to poor prognosis and aggressive disease progression [59,60,61,62,63,64]. Some studies show that the inhibition of ALDOA, using GalNAc-siRNA or small molecule inhibitors, effectively suppresses cancer metabolism in HCC and PCa, suggesting that targeting ALDOA may be a promising target for treating cancer [61,64].

Glyceraldehyde-3-phosphate dehydrogenase (GAPDH) is one of the most essential enzymes concerning cell energy metabolism [65]. It’s a glycolytic enzyme responsible for catalyzing rate-limiting sixth step in glycolysis, which is the reversible conversion of glyceraldehyde-3-phosphate (G-3-P) to 1,3-diphosphoglycerate. GAPDH is overexpressed in cancers and involved in many cancerous cellular processes, such as regulating apoptosis and tumor progression. Therefore, inhibiting GAPDH can play a significant role in cancer treatment, especially in treating tumor cells resistant to traditional therapy [65,66,67,68]. However, a small number of inhibitors have not yet been studied. One of the most promising GAPDH inhibitors is 3-bromopyruvate, which targets GAPDH enzymatic activity mainly. In preclinical studies, ME3BP-7, a microencapsulated form of 3-BP, was tested for its efficacy in treating pancreatic cancer (PDAC). In in vitro experiments, ME3BP-7 demonstrated strong cytotoxicity against PDAC cells overexpressing MCT1, effectively inhibiting growth and inducing cell death. In in vivo PDAC mouse models, ME3BP-7 significantly reduced tumor growth and metastasis without causing toxicity, unlike free 3BP, which resulted in severe side effects [69]. One study identified DC-5163 as a novel GAPDH inhibitor that led to cell apoptosis, decreased glucose uptake, and lactic acid production of cancer cells, so it can be a promising novel anticancer therapeutic [70]. Unfortunately, there are limitations concerning this therapy because inhibiting GAPDH can lead to unknown adverse events, so further investigation is pivotal to using this in practice [71].

The pentose phosphate pathway (PPP) interacts with glycolysis and is a key factor in supporting cancer metabolism. PPP is an excellent source of nicotinamide adenine dinucleotide phosphate hydrogen (NADPH), which protects cancer cells from oxidative stress. Moreover, PPP produces metabolites necessary for lipid and nucleotide synthesis, such as ribose-5-phosphate (R5P), essential for synthesizing nucleotides, especially important for rapidly proliferating cancer cells. To conclude, PPP provides the metabolites necessary for cell growth, DNA repair, and adaptation to the harsh conditions of the tumor microenvironment.

Glucose-6-phosphate dehydrogenase (G6PD) is the rate-limiting enzyme that catalyzes the key NADPH-generating reaction. G6PD is overexpressed in cancers such as breast, liver, colorectal cancer, and leukemia. Studies have shown that its overexpression is associated with a poorer prognosis and greater aggressiveness of these cancers. Based on this information, G6PD inhibition is a promising therapeutic target in anticancer therapy. Potential G6PD inhibitors such as 6-aminonicotinamide (6-AN), dehydroepiandrosterone (DHEA), polydatin, and RRx-001 are already being investigated, and results are showing that within these inhibitors, some have high efficacy, good tolerability, and overall promising effects. For instance, RRx-001 can be used as a chemo- and radiosensitizer, enhancing the effectiveness of cancer therapy while protecting healthy tissue from treatment toxicity. It has undergone phase 1 and 2 trials, showing potential in treating cancers, including lung cancer, melanoma, and colorectal cancer. Administered in combination with chemotherapy and radiotherapy improved tumor response while reducing toxicity through activation of Nrf2 pathway. Further phase 3 studies are needed to confirm its clinical efficacy [71,72,73,74,75].

Glutamine (Gln) is a nonessential amino acid (NEAA) that can be synthesized in the human body by the glutamine synthesis enzyme (GS). Gln is predominantly used in synthesizing nucleotides, lipids, amino acids, and other pathways such as gluconeogenesis, the TCA cycle, and glutathione metabolism. Thanks to these properties, enhancing glutamine metabolism is beneficial for cancer growth and progression. This can be achieved by many mechanisms, such as increased glutamine transport in lung, breast, colorectal, pancreatic, and head and neck cancer cells or increased glutaminolysis in breast, colorectal, lung, prostate, and pancreatic cancer cells. The reaction catalyzed by glutaminase (GLS) is the first rate-limiting step in glutaminolysis and, therefore, is an attractive target for anticancer therapy. GLS inhibitors, such as CB-839 and BPTES, are currently being investigated. The results showed attenuation of cancer growth and proliferation in multiple cancer cells, but simultaneously the activation of alternative metabolic pathways enabling cancer cells to survive. This draws attention to the potential utilization of GLS inhibitors in combination therapy as a novel approach to treating glutamine-dependent cancers because of their insufficient impact on their own [76,77,78,79]. Studies already show promising results in combining targeting glutamine metabolism with conventional therapy [80].

Phosphoglycerate dehydrogenase (PHGDH) is an enzyme that plays a significant role in the serine biosynthesis pathway and converts 3-phosphoglycerate into 3-phosphohydroxypyruvate. This pathway supports nucleotide synthesis, methylation, and NADPH production, enabling cancer cells to grow and protect them against oxidative stress. PHGDH overexpression is frequently observed in breast, lung, colorectal, melanoma, and pancreatic cancers, where it promotes tumor progression, aggressiveness, and poor prognosis by supporting pathways critical for cancer proliferation. It was investigated that heterogeneous PHGDH expression in breast tumors correlates with longer metastasis-free survival, while higher expression correlates with worse outcomes. Conversely, the knockdown of PHGDH inhibits tumor growth and sensitizes cells to ferroptosis, which makes targeting PHGDH therapeutically relevant [39,81,82,83]. Studies of PHGDH inhibitors, such as CBR-5884, show antitumor efficacy in breast and epithelial ovarian cancers both in vitro and in vivo [39,82,84]. Based on the former statements, we can acknowledge that targeting PHGDH is a promising strategy for developing novel cancer therapies.

Dichloroacetate (DCA) is an organic compound that is an analogue of acetic acid [85] It is an inhibitor of pyruvate dehydrogenase kinase (PDK) and its isoforms [86].The inhibition of PDK results in the inability to phosphorylate and, at the same time, inhibit the activity of pyruvate dehydrogenase (PDH), which is an enzyme converting pyruvate to acetyl-CoA—this reaction is the step that connects glycolysis and the Krebs cycle. Increased PDH activity in cancer cells disrupts their metabolism, because the input of anaerobic glycolysis is reduced and the contribution of oxidative phosphorylation is increased. In addition, mtDNA mutations, often occurring in tumorigenesis, additionally increase the dysfunction of the electron transport chain (ETC) and reduced lactate production counteracts the acidic TME. The effect of these actions is the inhibition of tumor growth and promotion of its death [85,86,87].

The use of DCA in anticancer therapy is a popular topic. Many in vitro and in vivo studies are being conducted. The efficacy of DCA is being studied in combination therapies with another anticancer drug and/or with radiotherapy. For example, a study was conducted using cisplatin-based chemoradiotherapy (CRT) in combination with DCA in 21 patients, whereas 24 patients received placebo. All patients had locally advanced head and neck squamous cell carcinoma (LA-HSNCC) [88]. At the end of treatment complete response rates were significantly higher in the DCA group than in the placebo group; however, survival outcomes were not significantly different between groups. Another clinical case is a 57-year-old female patient with stage 4 colon cancer who underwent oral DCA therapy. As a result of this therapy, the tumor stabilized for 4 years. Therefore, DCA affects the stability of cancer in advanced stages [89].

**Table 1:** Examples of compounds targeting cancer metabolism.

Enzymes	Their Inhibitors with Anti-Cancer Properties	References
PKM2	shikonin metformin proanthocyanidin B2 curcumin resveratrol	[44,45,46,47,50]
HK2	lonidamine (LND) metformin 2-deoxyglucose (2-DG) 3-bromopyruvate (3-BrPA) VDA-1102	[51,52,53,54,56,57,58]
ALDOA	GalNAc-siRNA	[59,60,61,62,63,64]
GAPDH	3-bromopyruvate (3-BrPA) DC-5163	[65,66,67,68,70,71]
G6PD	6-aminonicotinamide (6-AN) dehydroepiandrosterone (DHEA) polydatin RRx-001	[71,72,73,74]
GLS	CB-839 BPTES	[76,77,78,79]
PHGDH	CBR-5884	[39,82,84]
PDK	dichloroacetate	[85,86,87,88,89]

### 4.2. Impact of Metabolic Therapies on Cancer Resistance

Resistance to cancer therapies is a phenomenon that frequently leads to therapy failure and, despite enormous progress in treating cancer, remains a significant difficulty to overcome [72,90,91]. Resistance to cancer treatment can be divided into two groups: intrinsic resistance, which exists even before administering medication, and acquired resistance, which develops during or after treatment. Resistance is mainly caused by metabolic reprogramming, cancer adaptation to its microenvironment, and activation of HIF-1α. Most lethal cancers, like breast or gastric cancer, exhibit resistance to chemotherapeutic agents, including doxorubicin, cisplatin, docetaxel, paclitaxel, and 5-fluorouracil [44,72,74,90]. Consequently, chemoresistance is a significant cause of patient death, and finding a way to overcome it is crucial to properly treating cancer in the future [90,91].

Many studies show that metabolic therapies are a promising potential target for affecting anticancer drug sensitivity and overcoming resistance. For instance, lung, breast, and ovarian cancer frequently develop resistance to cisplatin, but studies showed that targeting cancer cell metabolism by inhibiting glutathione synthesis makes cancer cells more sensitive to cisplatin treatment [91]. Additionally, inhibiting PKM2 can also overcome cisplatin resistance in many cancer cells [44]. Another study shows that inhibiting G6PD increases the efficacy of cancer treatment with cisplatin as well as paclitaxel [72]. Furthermore, inhibiting G6PD significantly increases resistant breast cancer cells’ sensitivity to doxorubicin [74].

Metabolic therapies are promising targets for enhancing the efficacy of existing treatments and overcoming resistance, and further research must be implemented in practice.

Unfortunately, cancer cells can also develop resistance to antimetabolic drugs. They exhibit significant metabolic flexibility, so when glycolysis is blocked, cells can shift to alternative energy pathways, such as increasing oxidative phosphorylation (OXPHOS) activity. Moreover, during metastasis, tumor cells often alter their metabolic profiles, reducing the efficacy of therapies targeting the metabolism of the primary tumor. Another mechanism of resistance involves the activation of alternative pathways, such as the upregulation of amino acid transporters, which allows cells to bypass therapeutic blockades. In some cases, treatment can lead to the reactivation of previously suppressed metabolic pathways, enabling tumor cells to survive despite limited access to essential resources. Additionally, anti-metabolic drugs can influence the tumor microenvironment, including immune cells, which weakens the immune response and further reduces treatment efficacy. An example of cancer cells overcoming antimetabolic therapy and continuing proliferation is the survival of breast cancer cell lines after the administration of GLS inhibitors (BPTES and CB-839), where the cells utilize pyruvate as an alternative anaplerotic substrate. This prevents a decrease in fumarate levels in the TCA cycle [79,92].

## 5. Challenges and Future Directions

Many challenges in targeting cancer metabolism include metabolic heterogeneity, metabolic adaptability, interactions with TME, and adverse events. Metabolic heterogeneity results from diverse tumor microenvironment conditions and genetic mutations [84,91,93]. It relies on the statement that no matter what histopathological type the tumor is, every single tumor has its distinct metabolic phenotype (intertumoral heterogeneity), and changes in metabolism can even occur within the same tumor mass (intratumoral heterogeneity). According to TNBC, intertumoral heterogeneity can be divided into three metabolic subtypes with various sensitivities to glycolysis and fatty acid synthesis inhibitors. However, this classification has been widened over the years, and now we acquire more information about the metabolic heterogeneity of specific cancer types like HCC [39,84,91]. This factor shows that one metabolic drug can effectively work on some groups of cancer cells and have no impact on others, which impedes treating many cancers, including cutaneous neurofibromas (cNFs). It draws attention to the urgency of further investigating metabolism-based treatment strategies, which will enable choosing personalized metabolic therapy for each patient based on the metabolic type of the tumor [84,91,94].

Metabolic adaptability can occur during tumor progression or treatment. Metabolic adaptability depends on two main concepts: metabolic flexibility, the ability to use one nutrient as a substitute for another when one is lacking, and metabolic plasticity, which is the ability to use one nutrient in many ways in case one pathway is blocked. It allows cancer cells to adapt to harsh conditions. One example is adjusting to a low-oxygen environment due to HIF-1, increasing glycolytic flux [39]. Secondly, cancer cells can adapt to a new environment. For instance, in breast cancer, cells migrating from the primary tumor to a metastatic location can temporarily increase their OXPHOS activity. Moreover, metastatic cells, experiencing harsh environmental conditions and adapting to them, become more aggressive. Consequently, metastasis, not primary tumors, is the main cause of death in patients with cancer. This indicates that treating metastatic tumors may require different methods than treating primary tumors. In addition to adapting to a new location, cancer cells can change their metabolism to develop drug resistance. This shows how crucial it is to stay updated with metabolic changes in cancer cells to use proper treatment strategies [84,91].

Another factor is the possibility of targeting tumors and TME elements, such as immune cells, during therapy. It can cause an impairment of the immune system through many mechanisms, such as inhibiting glucose transporters, which can hurt macrophages.

The last factor limiting the efficacy of cancer treatment, discussed in this review, is the difficulty in developing therapeutics that simultaneously suppress cancer cells and have no toxicity to normal cells [84,95]. Many anticancer drugs, including doxorubicin, cisplatin, 5-fluorouracil, and methotrexate, are toxic to normal cells, and after treatment, patients have to deal with many side effects [96,97,98,99,100,101,102].

## 6. Conclusions

Understanding the metabolic reprogramming of cancer cells is a crucial and indispensable aspect of optimizing therapeutic strategies. Furthermore, it is fundamental to developing novel and more effective approaches to cancer treatment methods. The key processes undergoing alterations in cancer cells include the metabolism of glucose, fatty acids, and glutamine, as well as pathways that modify the tumor microenvironment. These changes enable cancer cells to fulfill their heightened energetic and biosynthetic demands, support continued growth and progression, and confer a competitive advantage in substrate acquisition over surrounding cells. Given the critical role of the previously mentioned metabolic alterations, many novel therapies are based on inhibiting enzymes that play a pivotal role in those processes. Efforts are focused on developing targeted therapies that selectively act on cancer cell-specific enzymes while minimizing the impact on the host’s healthy cells.

Additionally, a strong emphasis is on personalizing treatment by tailoring it to the specific metabolic phenotype of an individual patient’s tumor. However, a significant challenge occurs regarding cancer cells’ metabolic plasticity, enabling them to acquire resistance to therapeutic interventions. In conclusion, understanding the metabolic alterations occurring in cancer cells is fundamental to effective treatment, highlighting the critical need for ongoing research to clarify the mechanism of metabolic reprogramming, as such knowledge is essential for advancing cancer treatment outcomes and reducing the likelihood of recurrences.

## Data Availability

Most of the original data presented in the study are openly available in online searches.
